# The Voluntary Hospitals' Budget

**Published:** 1915-06-12

**Authors:** 


### THE VOLUNTARY HOSPITALS' BUDGET.

Hospital Sunday has again come round, bringing with it the necessity for considering ways and means, but this year the circumstances of the time we are living in are of a totally different character from those which were current when we discussed the 1914 budget of the Metropolitan Hospitals and Dispensaries in our last Hospital Sunday Special Number. Then we appealed for gifts out of the fullness of the purses of the charitable; now, when everyone is struggling under the ever-increasing burden of the war, our appeal to them must be: Out of your smaller spending power make your gifts to the medical charities at least what they used to be, for the necessities of these charities are infinitely greater to-day than they were a year ago. With still greater emphasis than ever, too, do we appeal to those who have not in the past extended their gifts to this form of charity. Now is their opportunity to come to the assistance of those who hitherto have shouldered the burdens of the voluntary charities, and, by providing a new field from which hospital managers can look for revenue, supplement the efforts of their fellow-citizens who have kept up the voluntary system for the benefit of all.

The ordinary expenditure of the Metropolitan Voluntary Hospitals and Dispensaries is increasing by about £40,000 a year. In 1911 it was about £1,215,000; in 1912, about £1,255,000; and in 1913, about £1,295,000. In 1915, therefore, it will certainly be, on a very conservative estimate, not less than £1,300,000, strive though the managers may to cut down expenses in every possible direction. And to meet this expenditure the hospitals and dispensaries have to rely upon three main sources of revenue.

First and most important of these are the gifts of the charitable for which we are now appealing, such as subscriptions, donations, entertainments, amounts raised by the King's Fund and the Hospital Sunday and Saturday Funds, and other receipts of this character. This source of revenue, together with the payments made by patients for treatment received, supplies over half the amount required for the upkeep of the hospitals, and may be taken as worth about £750,000 a year. Whether it will reach that figure in 1915 is a matter which now depends to a great extent upon the possibility of the old supporters continuing to give as they have done in the past, and upon new givers flocking in to fill up the gaps which may be caused by smaller subscriptions and donations being sent by former givers. £750,000 is the equivalent of 11s. 6d. towards every sovereign of the anticipated expenditure of £1,300,000.

Secondly, there is the income from investments and property. The value of this is increasing by about £10,000 a year, and should continue to do so. The amount received in 1913 was £350,000; so that in 1915 about £370,000 should be received under this head, or about 5s. 8d. towards each sovereign of the estimated expenditure for that year.

These two fairly certain sources of income may therefore be looked on as providing 17s. 2d. towards every £1 to be expended, and there only remains one other quarter from which revenue can be derived, viz. the gifts left to hospitals in the shape of legacies. These naturally vary in amount from year to year, having been during the last ten years as low as £186,000 (in 1906), and as high as £424,000 (in 1913). The average for the ten years (1904-1913) has been £320,000. Assuming legacies in 1915 reach the average of the last ten years, it will be necessary to take £180,000, or nearly three-fifths, of the legacies to be expected, in order to meet the estimated expenditure for the year. In reality, however, legacies should be looked upon as of the nature of extraordinary income, and should be utilised to assist in meeting the expenditure required for rebuilding, for improvements necessitated by the rapid march of science, or for extensions where overcrowding has become apparent, all of which items of extraordinary expenditure have now to be met by special appeals to those who already give largely.

Extraordinary expenditure we have entirely left out of consideration in our estimates as we are more concerned to-day with what we have to do to meet the ordinary every-day needs of the hospitals. It may, perhaps, be as well, however, to mention here that a further sum of from £200,000 to £300,000 is found each year under this head to meet the special needs which arise from time to time in looking after the care of the sick of the Metropolis.

In conclusion, therefore, we ask the old supporters of the Metropolitan Hospitals and Dispensaries to give this year, as nearly as possible, as liberally as they have done in the past; and we call on all those who have not hitherto given to remember what the voluntary hospitals have done, and are doing, for the entire community, and to become supporters of them at a time when it seems possible these charities may have to suffer in consequence of the heavy calls being made on the rest of the community.

Table Showing the Sources of the Income of the Metropolitan Hospitals and Dispensaries in the Ten Years 1904 to 1913.

| Year | From the Living | | From the Dead | | | | Total Income |
|---|---|---|---|---|---|---|---|
| | Subscriptions, Donations, Patients' payments, etc. | Percentage of Total | Investments | Percentage of Total | Legacies | Percentage of Total | |
| | £ | | £ | | £ | | £ |
| 1904 | 603,425 | 50 | 258,567 | 22 | 319,303 | 28 | 1,181,295 |
| 1905 | 610,130 | 49 | 264,790 | 21 | 373,914 | 30 | 1,248,834 |
| 1906 | 635,377 | 58 | 272,704 | 25 | 186,284 | 17 | 1,094,365 |
| 1907 | 653,320 | 48 | 296,218 | 22 | 407,918 | 30 | 1,357,456 |
| 1908 | 712,461* | 56 | 290,491 | 23 | 265,493 | 21 | 1,268.445 |
| 1909 | 701,458 | 57 | 294,206 | 24 | 240,523 | 19 | 1,236,187 |
| 1910 | 703,841 | 50 | 309,320 | 22 | 394,478 | 28 | 1,407,639 |
| 1911 | 738,622 | 53 | 329,253 | 24 | 322,949 | 23 | 1,390,824 |
| 1912 | 741,439 | 55 | 338,425 | 25 | 272,090 | 20 | 1,351,954 |
| 1913 | 845,908* | 52 | 349,295 | 22 | 424,339 | 26 | 1,619,542 |

* Includes £72,565 and £103,970, the amounts received by the London Hospital in 1908 and 1913 respectively as the result of its Quinquennial Appeals in those years.

